# Reactivity of Resorcinol on Pt(511) Single-Crystal Surface and Its Effect on the Kinetics of Underpotentially Deposited Hydrogen and Hydrogen Evolution Reaction in 0.1 M NaOH Electrolyte

**DOI:** 10.3390/molecules29133220

**Published:** 2024-07-07

**Authors:** Bogusław Pierożyński, Mateusz Kuczyński, Tomasz Mikołajczyk, Piotr Sołowiej

**Affiliations:** 1 Department of Chemistry, Faculty of Agriculture and Forestry, University of Warmia and Mazury in Olsztyn, Łódzki Square 4, 10-727 Olsztyn, Poland; mateusz.kuczynski@uwm.edu.pl (M.K.); tomasz.mikolajczyk@uwm.edu.pl (T.M.); 2 Department of Electrotechnics, Energetics, Electronics and Automatics, University of Warmia and Mazury in Olsztyn, Oczapowskiego 11 Street, 10-736 Olsztyn, Poland; pit@uwm.edu.pl

**Keywords:** resorcinol, RC ion, electroreduction of RC ion, UPD H, HER, Pt(511) single-crystal plane, *ac*. impedance spectroscopy

## Abstract

This article presents cyclic voltammetry, Tafel polarization, and *ac*. impedance spectroscopy examinations of resorcinol (RC) ion reactivity on Pt(511) single-crystal plane and the effect of surface-electrosorbed RC ions on the kinetics of UPD H (underpotentially deposited hydrogen) and HER (hydrogen evolution reaction) processes in 0.1 M NaOH solution. Obtained data delivered a proof for the RC ion surface adsorption and its later electroreduction over the potential range characteristic for the UPD H. A favourable role of platinum-adsorbed resorcinol anions on the kinetics of the UPD H and HER processes is also discussed. The above was explained via the recorded capacitance and charge-transfer resistance parameters (the presence of resorcinol at 1.5 × 10^−3^ M in 0.1 M NaOH caused significant reduction in the resistance parameter values by 3.9 and 2.6 times, correspondingly, for the UPD of H at 50 mV and the HER process, examined at −50 mV vs. RHE) along with the charge transients, produced by injecting small amounts of RC-based 0.1 M NaOH solution to initially RC-free base electrolyte on the Pt(511) electrode plane (a large cathodic charge-transient density of −90 µC cm^−2^ was recorded at the electrode potential of 50 mV).

## 1. Introduction

Remarkable catalytic properties of platinum-based catalysts, including well-ordered single-crystal Pt planes [1,2], commenced extensive research activities into the electrochemical performance of various organic molecules. The current article is based on previously published studies from this laboratory on the adsorption and electrochemical reactivity of simple organic molecules, including guanidine, acetamidine, formamidoxime, and urea, with Pt single-crystals [3,4,5,6,7], as well as recently published articles on resorcinol (RC) at polycrystalline Pt electrode [8,9], in both H_2_SO_4_ and NaOH supporting electrolytes.

The current study includes the electrosorption and further electrochemical reduction process of resorcinol ions at the surface of Pt(511) plane [also denoted as 3(100) × (111), which refers to (100) terraces of three atomic rows wide, separated by monoatomic steps having (111) geometry] in 0.1 M sodium hydroxide solution [10,11]. It should be stressed that the use of highly oriented Pt single-crystal surfaces is very important as it allows the characterization of the relationship between the catalyst’s surface structure (e.g., the width of terraces) and its reactivity, also in relation to mutual interactions of, e.g., surface adsorbed reaction intermediates. In the adsorbed state, resorcinol ions (or the products of their electroreduction) had a significant influence on the kinetics of underpotentially deposited hydrogen (UPD H) and later HER (hydrogen evolution reaction) processes.

The UPD of H emerges at potentials positive to the H_2_ reversible potential and is observed for some semi-noble and noble metals, including Pd, Rh, Pt, and Ir (Equation (1)) illustrates the process of UPD of H for Pt in alkaline medium, with a water molecule as the source of proton). Then, as the H_2_ reversible potential is exceeded, the HER becomes initiated, typically via the Heyrovsky electrochemical desorption step (Equation (2)) [12,13,14,15].

(1) $$H_{2}O+Pt+1e^{-}\;\;↔\;\;Pt-H_{ads}+OH^{-}$$

(2) $$Pt-H_{ads}+H_{2}O+1e^{-}\;\to \;H_{2}↑+OH^{-}+Pt$$

Furthermore, resorcinol is broadly used by industry in the production of many organic compounds and synthetic materials, also rubber. Then, the presence of water-soluble phenolic species, such as resorcinol, might have a substantial effect on the performance of operating water electrolyser and fuel cell units. This is because the RC molecules (or ions) under suitable external conditions could become susceptible to catalytic surface-adsorption and electrooxidation processes (especially valid for noble and semi-noble metals and their alloys). Then, under such unfavourable circumstances, the RC-modified catalyst’s reactivity (for instance, Pt for hydrogen oxidation or H_2_ evolution reaction) could considerably be altered [16,17,18]. These aspects are of superior technological importance, as renewable energy-based hydrogen economy slowly becomes our life’s reality. Of special economical significance are hydrogen production systems based on water electrolysers powered by high efficiency photovoltaic panels (see Ref. [19] for details).

## 2. Results and Discussion

### 2.1. Analysis of Cyclic Voltammetry, Tafel Polarization, and Charge-Transient Results

**Figure 1:**
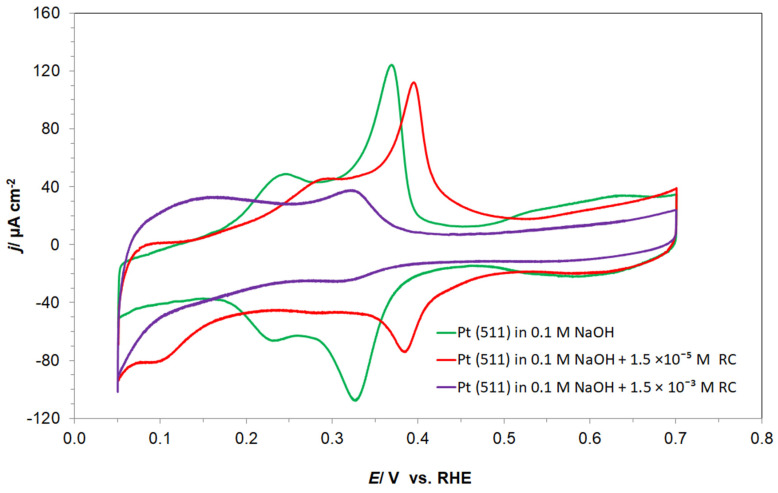
CV profiles for Pt(511) surface, registered over the potential span typical to UPD H adsorption in 0.1 M NaOH solution (on the second cycle), at a scan rate of 50 mV s^−1^, in the absence and presence of RC, at the indicated concentrations.

The cyclic voltammetric (CV) performance of Pt(511) surface, in pure and the RC-modified (at 1.5 × 10^−3^ M) 0.1 M NaOH solution, is illustrated in Figure 1. Thus, in the absence of RC, the CV profile of the Pt(511) single-crystal plane in 0.1 M NaOH is primarily characterized by the presence of a sharp peak at ca. 0.38 V in the positive scan (and at 0.33 V in the negative scan), which corresponds to the reversible process of H adsorption on the (100) terrace sites. The mean electric charge measured between 0.05 and 0.70 V vs. RHE, after subtracting the double-layer charge contribution, came to 226 µC cm^−2^.

**Scheme 1:**
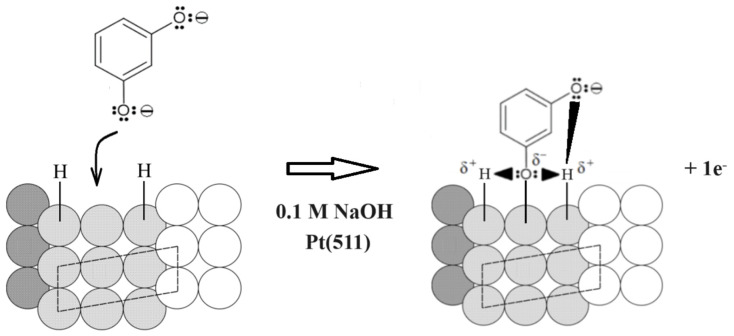
The proposed mechanism of RC ion electrosorption on the surface of Pt(511) electrode in 0.1 M NaOH solution along with RC ion-to-H attractive interaction in the adsorbed state for the potential span covering UPD of H.

After the introduction of resorcinol into the supporting solution, the total voltametric charge (see a blue curve in Figure 1) became radically reduced by about 40% to 136 µC cm^−2^. The latter is strongly believed to result from concurrent Pt surface co-adsorption and interaction of large resorcinol ions, formed after complete RC ionization under alkaline environment [20], with UPD hydrogen atoms (see the proposed mechanism for the resorcinol oxidative Pt electrosorption and the RC ion-to-H attractive interaction, i.e., via partial or complete negative charge on oxygen atom vs. partial positive charge on hydrogen atom, in Scheme 1). The above is also later validated through the recorded *ac*. impedance behaviour, described in detail in Section 2.2 below.

Furthermore, for a fundamentally reduced RC ion concentration of 1.5 × 10^−5^ M (see a red curve in Figure 1), the UPD of H adsorption/desorption regions are more pronounced, as those recorded at a high resorcinol concentration, where the observable sharp H adsorption and desorption peaks are significantly (*ca*. 30–50 mV) displaced towards anodic electrode potentials. Most importantly, an explicit voltametric reduction feature, observed in the presence of RC over the potential range of 0.05–0.15 V vs. RHE (Figure 1), could tentatively be assigned to the process of the RC surface electroreduction.

**Figure 2:**
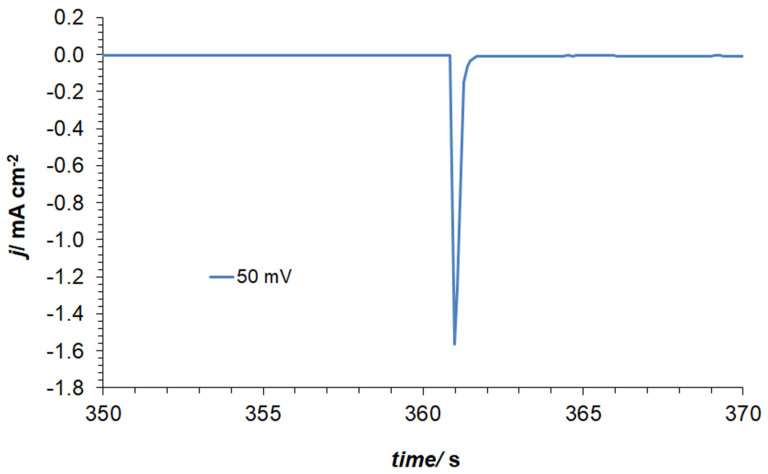
Current density vs. time charge transient recorded for electrode potential of 50 mV vs. RHE.

**Scheme 2:**
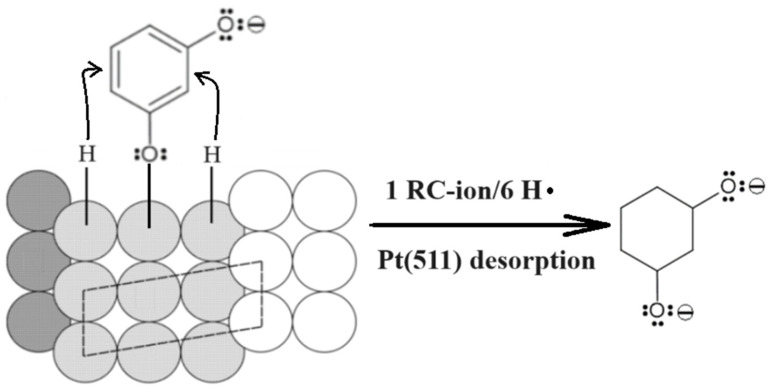
The proposed mechanism for resorcinol ion reduction to 1,3-cyclohexanediolate ion by UPD hydrogen on the surface of Pt(511) single-crystal plane in 0.1 M NaOH solution.

Hence, in order to further elucidate the above-described, non-negligible cathodic current feature (see again Figure 1), additional charge-transient experiments were performed at the electrode potential of 50 mV/RHE. As a result, a substantial, average cathodic charge-transient density of ca. −290 µC cm^−2^ was recorded (Figure 2), after subtracting the O_2_ reduction contribution (about −90 µC cm^−2^). Thus, no charge transient behaviour (beyond that involving unavoidable oxygen reduction, as small amounts of air are always being injected along with solution samples) was observed for injections of resorcinol-free 0.1 M NaOH samples. In brief, under the experimental conditions, the observed cathodic charge transients recorded at the potential value of 50 mV could only reflect the process of resorcinol ion electroreduction at the Pt(511) single-crystal surface, carried-out via the underpotentially deposited hydrogen radicals. The RC ion reduction process to form 1,3-cyclohexanediolate ion (see Refs. [21,22,23]) by UPD H atoms along with its further desorption that is proposed here is presented in Scheme 2 below.

**Figure 3:**
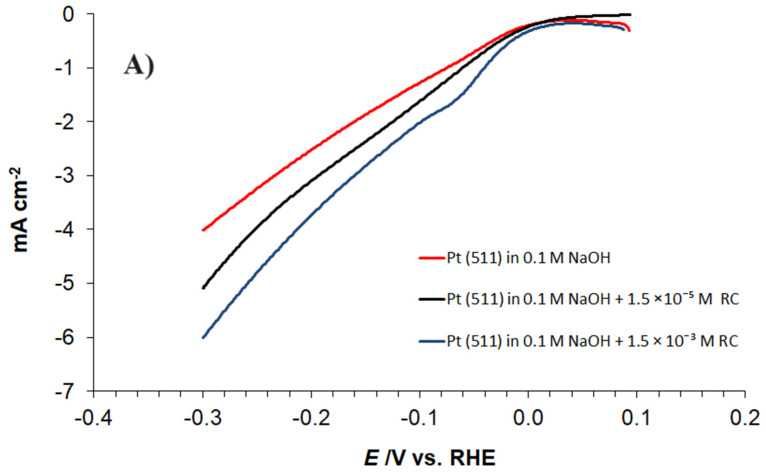

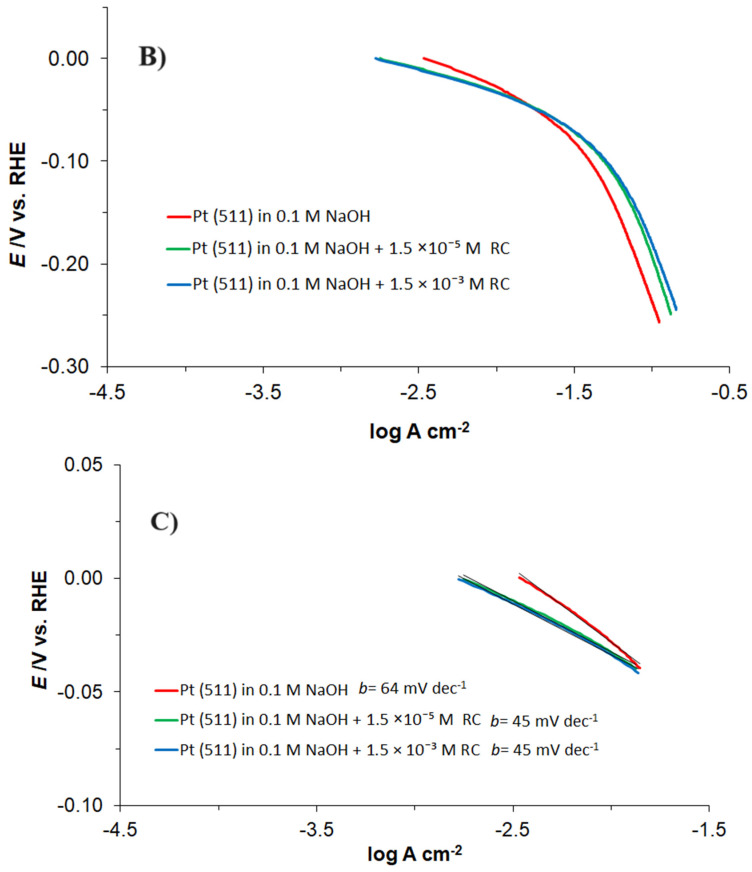
(**A**) Linear sweep voltammetry measurements for Pt(511) electrode surface, recorded over the potential range characteristic for HER (0.0 to −0.3 V vs. RHE) in 0.1 M NaOH supporting solution, at a sweep-rate of 50 mV s^−1^, in the absence and presence of resorcinol, at the concentrations indicated. (**B**) As above, but quasi-potentiostatic cathodic Tafel polarization curves for the HER (recorded at a rate of 0.5 mV s^−1^) with appropriate *iR* corrections made, based on the solution resistance derived from the impedance measurements. (**C**) As in (**B**), but showing low overpotential linear region with fitting lines along with calculated Tafel slopes.

Furthermore, supplementary linear-sweep voltammetry experiments, extended to the potential range negative to the hydrogen reversible potential (0.00 to −0.30 V/RHE), indicated that the Pt(511) surface-adsorbed RC species (or its electroreduction product) exhibited considerable, positive effect that was directly proportional to the RC concentration on the HER rates (Figure 3A). Hence, for the maximum overpotential value of 0.30 V, the recorded current densities were 1.3× and 1.5× greater for the RC ion concentrations of 1.5 × 10^−5^ and 1.5 × 10^−3^ M, correspondingly, than those derived in the absence of RC in the electrolyte.

A significant enhancement of the HER behaviour upon the introduction of resorcinol into the supporting electrolyte could also be observed over the kinetically controlled overpotential region of cathodic Tafel polarization curves (Figure 3B). For low overpotentials, the recorded cathodic Tafel slopes (parameter *b*) approached 64 and 45 mV dec^−1^ for the unmodified NaOH and the RC-modified solution, correspondingly (see Figure 3C). In addition, the Tafel-estimated values (over an initial overpotential range) of an exchange current density (*j*_0_) parameter approached 3.7 mA cm^−2^ (0.1 M NaOH), 1.8 mA cm^−2^ (0.1 M NaOH + 1.5 × 10^−5^ M RC), and 1.9 mA cm^−2^ (0.1 M NaOH + 1.5 × 10^−3^ M RC). The latter observation is in line with the literature-based *j*_0_ values recorded for Pt and platinum-derived hydrogen evolution reaction catalysts [24,25].

### 2.2. Analysis of ac. Impedance Results

**Figure 4:**
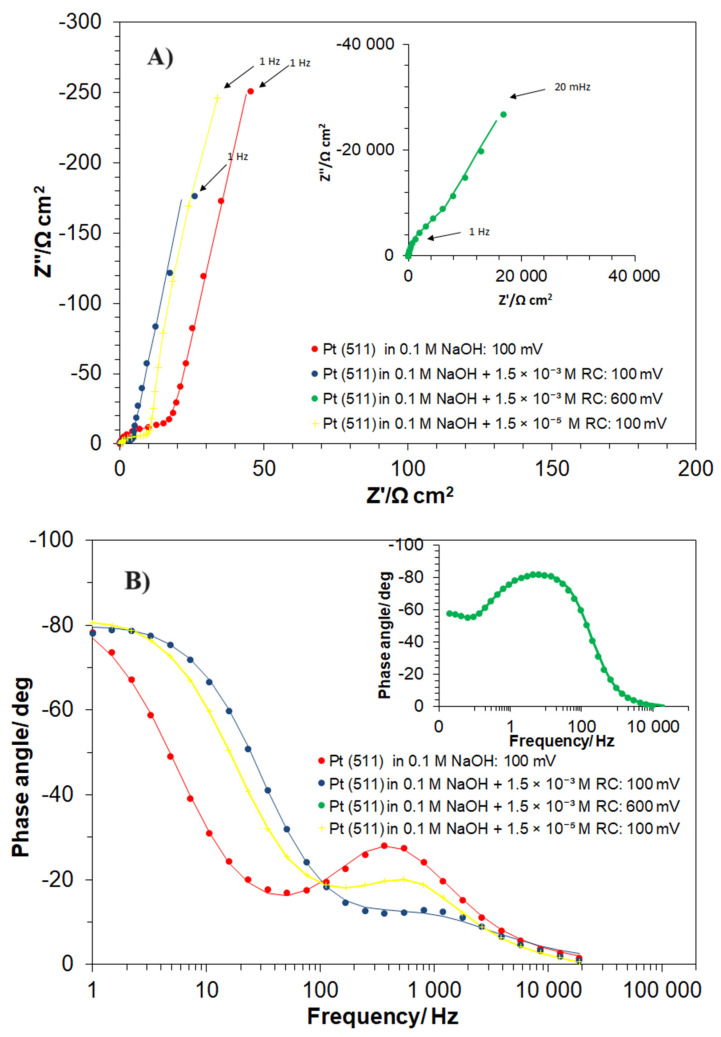

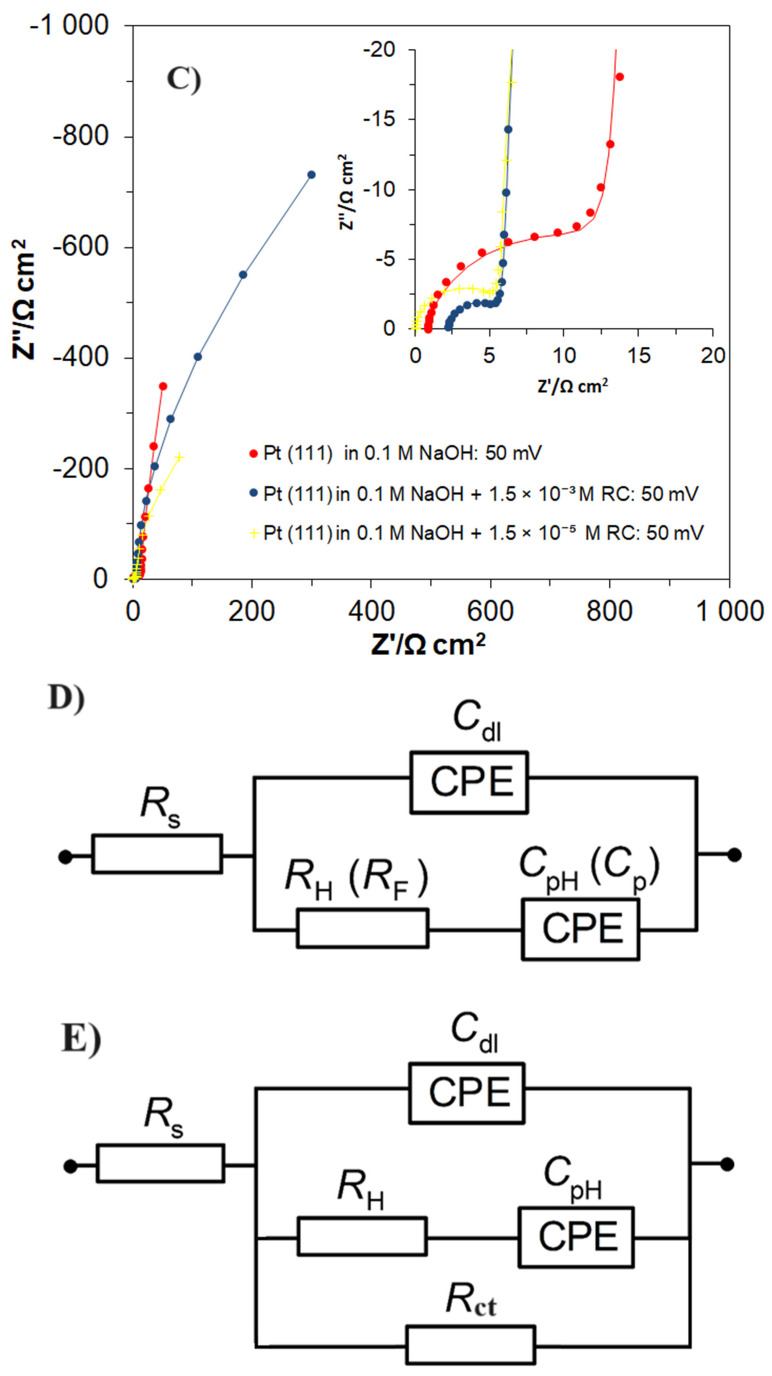

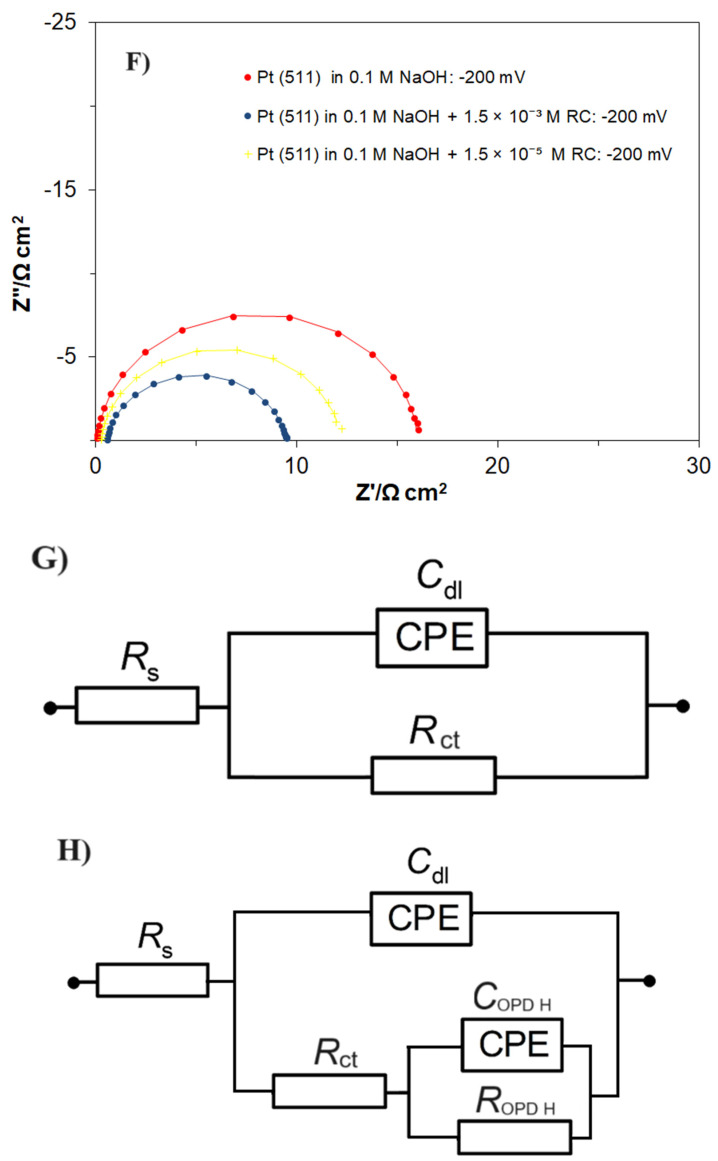
(**A**) Nyquist impedance plots for the processes of UPD of H and electrosorption of resorcinol anion on Pt(511) surface in contact with pure and RC-modified (at the concentrations of 1.5 × 10^−5^ and 1.5 × 10^−3^ M) 0.1 M sodium hydroxide solution, recorded at room temperature for the indicated electrode potentials. The solid lines correspond to the representation of the data according to the equivalent circuit shown in Figure 4D. (**B**) As above, but Bode phase-angle plots. (**C**) Nyquist impedance plots for the processes of UPD of H and electroreduction of resorcinol species on Pt(511) electrode surface in contact with 0.1 M NaOH, for the RC ion concentrations of 1.5 × 10^−3^ and 1.5 × 10^−5^ M, recorded at 293 K for the electrode potential of 50 mV. The solid lines correspond to the representation of the data according to the equivalent circuit shown in Figure 4E. (**D**) Equivalent circuit for UPD of H adsorption (or resorcinol anion adsorption) process, exhibiting a Faradaic pseudocapacitance, *C*_pH_ (or *C*_p_), charged via a Faradaic resistance, *R*_H_ (or *R*_F_), in a parallel combination with a double-layer capacitance, *C*_dl_, where both capacitance variables are CPE-modified, jointly in series with an uncompensated solution resistance, *R*_S_. (**E**) Equivalent circuit for UPD of H adsorption along with resorcinol ion electroreduction process, exhibiting a Faradaic pseudocapacitance, *C*_pH_, charged via a Faradaic resistance, *R*_H_, in parallel combinations with a double-layer capacitance, *C*_dl_ (both capacitance variables are CPE-modified), and a charge-transfer resistance, *R*_ct_ (responsible for RC ion reduction), jointly in series with an uncompensated solution resistance, *R*_S_. (**F**) Nyquist impedance plots for the process of HER on Pt(511) electrode surface in contact with 0.1 M NaOH, for the RC ion concentrations of 1.5 × 10^−3^ and 1.5 × 10^−5^ M, recorded at 293 K for the electrode potential of −200 mV. The solid lines correspond to the representation of the data according to the equivalent circuit shown in Figure 4G. (**G**) Equivalent circuit for the process of HER, exhibiting a Faradaic charge-transfer resistance, *R*_ct_, in a parallel combination with the CPE-modified double-layer capacitance, *C*_dl_, jointly in series with an uncompensated solution resistance, *R*_S_. (**H**) As above, but including a parallel combination of Faradaic pseudocapacitance, *C*_OPD H_, and resistance, *R*_OPD H_, parameters for overpotentially deposited (OPD) hydrogen species.

*Ac*. impedance electrochemical behaviour of the Pt(511) electrode in unmodified 0.1 M NaOH solution and in the presence of 1.5 × 10^−3^ M RC, examined over the potential range characteristic to the processes of underpotential deposition of hydrogen and electrosorption of resorcinol ion, is shown in Table 1 and Figure 4A–E. The process of UPD of H produces a semicircle present in the high and intermediate frequencies in the Nyquist impedance plot along with a straight line (Figure 4A), which defines typical capacitive behaviour, at relatively low frequencies. The latter quite often departs from a 90-degree angle, because of the widely described *capacitance dispersion* phenomenon in the literature [3,26], in relation to the catalyst’s heterogeneity and surface roughness effects.

Thus, the charge-transfer resistance, *R*_H_, parameter (corresponding to the inverse of the exchange rate for the UPD of H) radically increased from 14.1 (at 50 mV) to reach 149.8 Ω cm^2^ at 300 mV (where the rates of H adsorption become significantly slowed down). Then, the double-layer capacitance, *C*_dl_, values varied between 54 and 86 μF cm^−2^ for the same potential range. Furthermore, the maximum value of the hydrogen adsorption pseudocapacitance, *C*_pH_, parameter (around 700–730 μF cm^−2^) comes in line with the current density changes in the CV profile (see Table 1 and Figure 1 for details, and compare the *C*_pH_ behaviour recorded here to that previously presented for Pt(100) single-crystal plane in 0.5 M H_2_SO_4_ in Ref. [3]).

Then, introduction of RC (at the concentration of 1.5 × 10^−3^ M) into the supporting electrolyte resulted in the radically reduced *R*_H_ parameter values, i.e., 3.6 Ω cm^2^ at 50 mV (3.9 times) and 15.7 Ω cm^2^ at 300 mV (9.5 times), as compared to the resorcinol-free solution. Although the recorded *C*_dl_ and *C*_pH_ capacitance values generally resembled those of the RC-free NaOH solution, the high-potential (300 mV) *C*_pH_ value registered in the presence of resorcinol declined by nearly 70% (also, compare the cyclic voltametric profiles recorded in the absence and presence of RC in Figure 1). In fact, the occurrence of another partial semicircle over intermediate and low frequencies, at the potential of 50 mV (Figure 4C,E, and the respective *R*_ct_ value of 1416 Ω cm^2^ in Table 1), very well matches the voltammetric cathodic feature observed in Figure 1, over the potential range of 0.05–0.15 V. The latter is strongly believed to be linked with the Faradaic process of the RC ion electroreduction, achieved via surface-chemisorbed UPD H atoms (see Scheme 2 again), and complies with the results obtained through the charge-transient experiments discussed above and illustrated in Figure 2.

Nevertheless, contrary to the behaviour in the resorcinol-free solution, in the presence of RC, another partial semicircle becomes observed in the Nyquist impedance plots (over high and intermediate frequencies, and a low frequency capacitive line, at an inclination to the Z′ axis significantly lower than 90°). The above was identified over the potential range of 400–600 mV vs. RHE and could be seen as examples in a Nyquist impedance spectrum and a Bode phase-angle plot recorded at 600 mV in insets to Figure 4A,B, correspondingly.

The above-described behaviour is strongly believed to be associated with the Faradaic process of the RC ion electrosorption on the Pt(511) surface, where its kinetics are several orders in magnitude slower than those of the process of UPD of H (see the respective charge-transfer resistance, *R*_F_, and capacitance parameter values, *C*_p_ and *C*_dl_, recorded in Table 1 and Scheme 2 above for details). Then, in the adsorbed state, the RC ions and chemisorbed H atoms (with considerably limited surface coverage) undergo attractive interactions (Scheme 1 and Scheme 2), which as a result do significantly facilitate the kinetics of the underpotential deposition of H (again, compare the registered values of the *R*_H_ variable for the RC-free and resorcinol-modified NaOH solutions in Table 1). Furthermore, as for Pt single-crystal planes, the kinetics of the UPD H play “a precondition role” in the Faradaic process of bulk hydrogen evolution reaction (HER) [27,28], and the surface-adsorbed RC entity might potentially be considered as an efficient HER catalytic Pt surface conditioner.

Moreover, additional *ac*. impedance experiments carried out at fundamentally lower RC ion concentrations in the working electrolyte (1.5 × 10^−5^ M) provided kinetic UPD H data that are generally parallel (although the corresponding *R*_H_ parameter values are higher) with those obtained for higher RC concentration (also compare with the linear sweep voltammetry and Tafel polarization results, presented in Section 2.1 above). Interestingly, the RC reduction corresponding charge transfer resistance, *R*_ct_, parameter value (recorded at 50 mV vs. RHE, Table 1) comes to about 50% of that derived at higher RC concentration, which implies that the simultaneous accommodation of large number of resorcinol ions and hydrogen atoms on the (100) terraces is challenging and as such it significantly hinders the Pt-based RC ion surface electroreduction process. Simultaneously, the concentration of resorcinol anions seems to have a direct and important effect on the rate of their surface electrosorption (see Table 1 for details).

However, it has to be stressed that the above contrasts the conclusion of a recent report [8], i.e., analogous electrochemistry work on resorcinol, which covers polycrystalline Pt electrode examination in a 0.5 M H_2_SO_4_ solution. However, contrary to the behaviour in an acidic environment, under alkaline conditions, the surface electrosorption of the RC ions might be significantly restricted, due to the existing repulsive interactions, between already adsorbed resorcinol anions (see Scheme 1 and Scheme 2).

Supplementary *ac*. impedance data on the process of hydrogen evolution reaction, recorded in Table 2 and Figure 4F, provided additional support to the previously drawn conclusions that the presence of resorcinol species in sodium hydroxide baseline solution significantly facilitates the HER kinetics on the surface of Pt(511) single-crystal plane.

Hence, in the absence of RC, the charge-transfer resistance, *R*_ct_, parameter significantly decreased from 24.7 (at −50 mV) to 10.4 Ω cm^2^ at the electrode potential of −300 mV, whereas the double-layer capacitance, *C*_dl_, fluctuated between ca. 33 and 41 μF cm^−2^ for the same potential range. Then, the introduction of RC into the supporting solution resulted in fundamentally reduced *R*_ct_ values by 1.7 and 1.4 times (at −50 and −300 mV at the RC concentration of 1.5 × 10^−5^ M), and 2.6 and 1.6 times (at −50 and −300 mV at the RC concentration of 1.5 × 10^−3^ M), as compared to the resorcinol-free solution. At the same time, the charge-transfer resistance, *R*_OPD H_, parameter for overpotentially deposited [27] hydrogen (identified only at an initial overpotential value of 50 mV) also exhibited considerable reduction (from 5.1 through 3.2 to 2.8 Ω cm^2^), as the resorcinol concentration was raised from 0 to 1.5 × 10^−5^ to 1.5 × 10^−3^ M.

## 3. Materials and Methods

A 0.1 M sodium hydroxide supporting solution was prepared using high-quality NaOH pellets, procured from MERCK, and pure H_2_O, supplied by Direct-Q3 UV Merck-Millipore (Darmstadt, Germany) water purification system (18.2 MΩ cm H_2_O resistivity). The RC (Sigma-Aldrich, St. Louis, MO, USA, >99.0%) concentration in the working solution was of the order of 1.5 × 10^−3^ M (also, comparatively 1.5 × 10^−5^ M).

All electrochemical experiments were conducted with a three-partition Pyrex glass cell, which contained three electrodes: Pt(511) working electrode, aligned to the chosen crystallographic orientation with a back reflection, von Laue X-Ray diffraction method [29], with S_A_ ≅ 0.064 cm^2^, and palladium RHE, with a reversible hydrogen electrode, as a reference electrode (1.0 mm diameter, Sigma-Aldrich, 99.99%), and a counter electrode (CE) made from a 1.0 mm diameter coiled Pt wire (99.9998% purity, Johnson Matthey, Inc., Chicago, IL, USA). Prior to conducting the tests, the working solution was always deprived of oxygen with high-purity Ar (6.0, Linde), whose flow was also kept above the solution during the measurements.

*SP-240 Biologic Electrochemical System* (Biologic, Seyssinet-Pariset, France) was employed to conduct all electrochemistry experiments. *Ac*. impedance spectroscopy, Tafel polarization, and cyclic voltammetry (carried out at the scan rates of 0.5 mV s^−1^ and 50 mV s^−1^, respectively) tests were performed in this study. For the impedance measurements, the generator provided an output signal of 5 mV, whereas the frequency range was swept between 1.0 × 10^5^ and 1.0 Hz (or 0.020 Hz). The instrument was controlled by EC-Lab^®^ 10.4 software for Windows. Three impedance tests were conducted at each electrode potential with duplicability of such-obtained results of the order of 10%. The impedance data assessment was carried out with *ZView* 4.0 software for Windows, where the impedance spectra were fitted by the *LEVM* 6 program, written by J.R. Macdonald (see Ref. [30] for details).

In addition, charge-transient experiments were performed at the constant electrode potential mode through direct Pt surface injecting 100 µL amounts (Hamilton 710 N micro-syringe model) of ca. 1.0 × 10^−2^ M RC in 0.1 M NaOH (previously de-aerated by bubbling with high-purity Ar) for the initial resorcinol and oxygen-free sodium hydroxide solution. Five independent injection trials were carried out at the electrode potential of 50 mV vs. RHE (see Figure 2 for details).

## 4. Conclusions

The application of essential electrochemical techniques (ac. impedance spectroscopy, cyclic voltammetry, Tafel polarization, and chronoamperometric charge transient) to investigate the influence of resorcinol on the kinetics of UPD of H and HER processes, which were examined on Pt(511) single-crystal surface under an alkaline environment, provided the evidence of the substantial role that Pt-electrosorbed resorcinol anions played in the rates of the underpotential deposition of hydrogen (typically considered as a precursor to hydrogen evolution reaction for platinum-group catalysts) and hydrogen evolution reaction.

Nevertheless, additional laboratory studies will be required to attain the detailed understanding of the process of RC ion adsorption on the surface of the Pt(511) plane and to deliver insights into the competitive RC ion-to-UPD H adsorption (along with the further electroreduction of resorcinol ions by H radicals), especially on how it could affect the kinetics and mechanisms of the processes of UPD and OPD of H and later the Faradaic HER reaction.

## Figures and Tables

**Table 1:** Capacitance and resistance parameters for UPD of H and electrosorption of resorcinol ion at Pt(511) surface in contact with 0.1 M NaOH and in the presence of 1.5 × 10^−3^ and 1.5 × 10^−5^ M RC (registered at room temperature), obtained by fitting the equivalent circuits shown in Figure 4D,E to the produced impedance results (dimensionless φ parameter values for the CPE: constant phase element-modified circuits varied between 0.78 and 0.96; impedance replicability was typically below 10%; χ^2^ = 2 × 10^−5^ to 3 × 10^−3^).

*E*/mV	*R*_H_/Ω cm^2^	*C*_dl_/µF cm^−2^	*R*_ct_/Ω cm^2^	*C*_pH_/µF cm^−2^
0.1 M NaOH				
50	14.1 ± 0.2	69.8 ± 5.9	-	458 ± 7
100	25.2 ± 0.5	54.7 ± 3.6	-	291 ± 5
150	47.8 ± 0.8	53.6 ± 2.6	-	286 ± 5
200	71.5 ± 0.8	73.3 ± 2.0	-	446 ± 5
250	111.9 ± 1.5	85.7 ± 2.3	-	700 ± 13
300	149.8 ± 0.7	68.3 ± 0.7	-	731 ± 7
400	-	214.1 ± 2.6	-	
500	-	87.8 ± 2.8	-	
0.1 M NaOH + 1.5 × 10^−3^ M RC				
50	3.6 ± 0.1	38.6 ± 1.8	1416 ± 92	500 ± 39
100	5.1 ± 0.2	28.3 ± 7.2	-	454 ± 10
150	6.5 ± 0.1	38.0 ± 4.4	-	390 ± 10
200	8.5 ± 0.2	47.5 ± 5.4	-	351 ± 6
250	12.1 ± 0.3	51.3 ± 5.8	-	321 ± 7
300	15.7 ± 0.6	81.5 ± 12.0	-	231 ± 11
			*R*_F_/Ω cm^2^	*C*_p_/µF cm^−2^
400	-	93.1 ± 1.3	24,892 ± 2690	90 ± 13
500	-	70.3 ± 2.1	8454 ± 1502	73 ± 4
600	-	98.2 ± 1.2	9708 ± 1741	106 ± 13
0.1 M NaOH + 1.5 × 10^−5^ M RC				
			*R*_ct_/Ω cm^2^	*C*_pH_/µF cm^−2^
50	9.2 ± 0.6	39.9 ± 3.9	667 ± 9	599 ± 28
100	12.3 ± 0.8	37.3 ± 3.7	-	361 ± 17
150	24.7 ± 1.6	33.0 ± 8.2	-	197 ± 9
200	20.5 ± 1.3	37.3 ± 3.7	-	231 ± 11
300	49.5 ± 3.2	186.8 ± 18.5	-	335 ± 16
			*R*_F_/Ω cm^2^	*C*_p_/µF cm^−2^
400	-	91.3 ± 2.3	27,249 ± 165	123 ± 15
500	-	87.3 ± 5.5	4354 ± 58	89 ± 6
600	-	102.1 ± 3.8	5092 ± 68	211 ± 33

**Table 2:** Resistance and capacitance parameters for the process of HER at Pt(511) surface in contact with 0.1 M NaOH and in the presence of 1.5 × 10^−3^ and 1.5 × 10^−5^ M RC (registered at room temperature), obtained by fitting the equivalent circuits shown in Figure 4G,H to the produced impedance results (dimensionless φ parameter values for the CPE: constant phase element-modified circuits varied between 0.89 and 0.99; impedance replicability was typically below 10%; χ^2^ = 2 × 10^−7^ to 3 × 10^−5^).

*E*/mV	*R*_OPD H_/Ω cm^2^	*C*_OPD H_/µF cm^−2^	*R*_ct_/Ω cm^2^	*C*_dl_/µF cm^−2^
0.1 M NaOH				
−50	5.1 ± 0.2	401 ± 48	24.7 ± 0.3	33.8 ± 1.6
−100	-	-	17.4 ± 0.5	33.2 ± 3.3
−200	-	-	15.8 ± 1.0	41.5 ± 4.1
−300	-	-	10.4 ± 0.7	37.7 ± 3.7
0.1 M NaOH + 1.5 × 10^−5^ M RC				
−50	3.2 ± 0.2	89 ± 4	14.2 ± 0.7	38.8 ± 1.7
−100	-	-	11.6 ± 0.2	48.0± 2.8
−200	-	-	9.9 ± 0.6	57.0± 4.7
−300	-	-	7.5 ± 0.5	47.3± 5.1
0.1 M NaOH + 1.5 × 10^−3^ M RC				
−50	2.8 ± 0.1	419 ± 36	9.5 ± 0.5	42.2 ± 5.6
−100	-	-	8.9 ± 0.2	78.5 ± 3.7
−200	-	-	4.7 ± 0.1	41.7 ± 1.9
−300	-	-	6.5 ± 0.0	29.8 ± 1.4

## Data Availability

Data supporting reported results will be available upon request.
